# Supplementary material for: Splice-Junction-Based Mapping of Alternative Isoforms in the Human Proteome
Source: Cell Rep. Author manuscript; Available in PMC 2020 Jan 15. (PMC6961840; doi:10.1016/j.celrep.2019.11.026)
Supplement: 2 [file NIHMS1546469-supplement-2.zip › DF1/PXD006675/VentricularSeptum/VentricularSeptum_13_MYBPC3_AITQLLCETEGR.pdf]

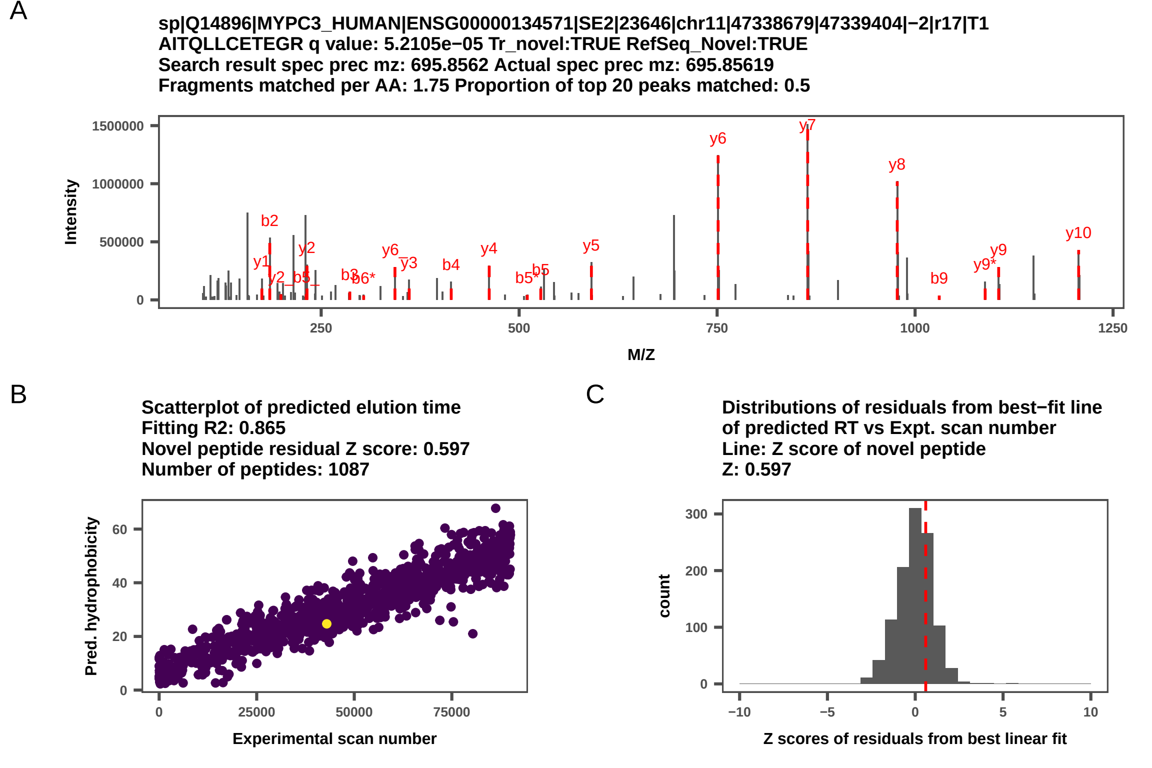
A
sp|Q14896|MYPC3_HUMAN|ENSG00000134571|SE2|23646|chr11|47338679|47339404|−2|r17|T1
AITQLLCETEGR q value: 5.2105e−05 Tr_novel:TRUE RefSeq_Novel:TRUE
Search result spec prec mz: 695.8562 Actual spec prec mz: 695.85619
Fragments matched per AA: 1.75 Proportion of top 20 peaks matched: 0.5
Intensity
1500000
1000000
500000
0
M/Z
250
500
750
1000
1250
b2
y1
y2
y2_
b5_
b3
b6*
y6_
y3
b4
y4
b5*
b5
y5
y6
y7
y8
b9
y9*
y9
y10
B
Scatterplot of predicted elution time
Fitting R2: 0.865
Novel peptide residual Z score: 0.597
Number of peptides: 1087
Pred. hydrophobicity
60
40
20
0
0
25000
50000
75000
Experimental scan number
C
Distributions of residuals from best−fit line
of predicted RT vs Expt. scan number
Line: Z score of novel peptide
Z: 0.597
count
300
200
100
0
−10
−5
0
5
10
Z scores of residuals from best linear fit
